# Inhibition of Hsp90 Augments Docetaxel Therapy in Castrate Resistant Prostate Cancer

**DOI:** 10.1371/journal.pone.0103680

**Published:** 2014-07-29

**Authors:** ShengYu Ku, Elena Lasorsa, Remi Adelaiye, Swathi Ramakrishnan, Leigh Ellis, Roberto Pili

**Affiliations:** 1 Genitourinary Program, Roswell Park Cancer Institute, Buffalo, New York, United States of America; 2 Department of Pharmacology and Therapeutics, Roswell Park Cancer Institute, Buffalo, New York, United States of America; 3 Department of Cancer Prevention and Pathology, Roswell Park Cancer Institute, Buffalo, New York, United States of America; 4 Department of Medicine, Roswell Park Cancer Institute, Buffalo, New York, United States of America; University of Texas Health Science Center, United States of America

## Abstract

First line treatment of patients with castrate resistant prostate cancer (CRPC) primarily involves administration of docetaxel chemotherapy. Unfortunately, resistance to docetaxel therapy is an ultimate occurrence. Alterations in androgen receptor (AR) expression and signaling are associated mechanisms underlying resistance to docetaxel treatment in CRPC. Heat shock protein 90 (Hsp90) is a molecular chaperone, which regulates the activation, maturation and stability of critical signaling proteins involved in prostate cancer, including the AR. This knowledge and recent advances in compound design and development have highlighted Hsp90 as an attractive therapeutic target for the treatment of CRPC. We recently reported the development of a MYC-CaP castrate resistant (MYC-CaP/CR) transplant tumor model, which expresses amplified wild type AR. Within, we report that a second generation Hsp90 inhibitor, NVP-AUY922, inhibits cell growth and significantly induces cell death in MYC-CaP/CR and Pten-CaP/cE2 cell lines. NVP-AUY922 induced proteasome degradation of AR, though interestingly does not require loss of AR protein to inhibit AR transcriptional activity. Further, NVP-AUY922 increased docetaxel toxicity in MYC-CaP/CR and Pten-CaP/cE2 cell lines *in vitro*. Finally, NVP-AUY922/docetaxel combination therapy in mice bearing MYC-CaP/CR tumors resulted in greater anti-tumor activity compared to single treatment. This study demonstrates that NVP-AUY922 elicits potent activity towards AR signaling and augments chemotherapy response in a mouse model of CRPC, providing rationale for the continued clinical development of Hsp90 inhibitors in clinical trials for treatment of CRPC patients.

## Introduction

Castrate resistant prostate cancer (CRPC) is an incurable and aggressive prostate cancer (PCa) phenotype. For several years, the primary treatment option of CRPC has been limited to docetaxel chemotherapy, which extends survival though for a limited time [1]. While the treatment of CRPC has been extremely challenging, the last 2 years has seen a dramatic change in therapeutic options for this disease. The second line cabazitaxel [2], the immunotherapy sipuluecel-T [3], the androgen synthesis inhibitor abiraterone acetate [4],the AR antagonist enzalutamide [5], and radium-223 [6] have all received recent FDA approval for the treatment of CRPC. Although these new therapies have expanded the treatments options for patients, sustainable suppression of CRPC growth still remains a primary challenge and new treatment strategies are still required.

Because docetaxel is an effective therapy, it still remains a critical component for first line treatment strategy of CRPC. Overcoming both acquired and intrinsic resistance to docetaxel has been a major clinical challenge and improving treatment outcomes for patients with docetaxel resistance is of high priority. With a greater understanding of underlying mechanisms of drug resistance, new therapeutic opportunities are emerging. This can create avenues for monotherapy treatment of docetaxel resistance CRPC or potentially re-sensitize CRPC patients to docetaxel therapy with novel combination strategies.

Targeting the androgen-AR axis with abiraterone acetate [4] and enzalutamide [5], as monotherapy, in patients with docetaxel resistant CRPC has demonstrated clinical benefit. While direct targeting of AR action is primarily desired, indirect targeting, via inhibition of AR associated proteins is an attractive approach. The molecular chaperone Hsp90 is a member of the heat shock protein family [7] and found to be overexpressed in multiple cancers, including PCa. Hsp90 has over 200 documented clients, including the AR, where Hsp90 prevents proteasome degradation and stabilizes AR conformation poised for ligand activation [7]. Because Hsp90 interacts with multiple client proteins, it is perceived that inhibition of Hsp90 could potentiate a greater catastrophic anti-tumor effect when compared to more direct targeted therapies. In pre-clinical studies, Hsp90 inhibition alone [8], [9], or more recently, in combination [10], [11] produced promising results in the treatment of PCa cells. Unfortunately, first generation Hsp90 inhibitors did not result in significant efficacy in clinical trials [12]–[14]. These initial Hsp90 inhibitors known as geldanamycin analogs, 17-allylamino-17 demethoxygeldanamycin (17-AAG) and 17-(dimethylaminotheyl-amino)-17-demethoxygeldanamycin (17-DMAG) were attributed to fail in the clinic due to poor solubility and pharmacokinetics, hepatotoxicity, susceptibility to P-glycoprotein efflux and lack of metabolism by NQO1/DT-diaphorase enzymes [15].

**Figure 1:**
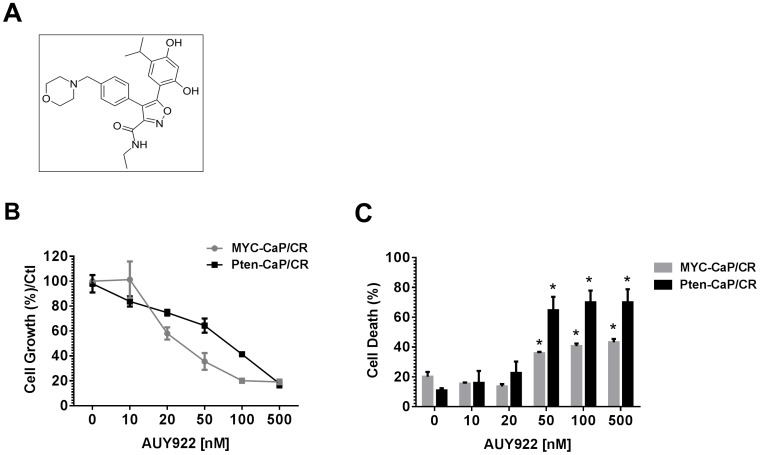
Castrate resistant prostate cancer cell line sensitivity to AUY922 treatment. (**A**) Chemical structure of NVP-AUY922. (**B**) Castrate resistant (MYC-CaP/CR and Pten-CaP/cE2) cell lines were treated with indicated concentrations of AUY922 for 48h. Adherent cells were fixed with 70% EtOH. Cell growth was assessed by staining fixed cells with crystal violet and measuring absorbance at 570 nm. Each point was normalized to the untreated control (0) and is represented by 3 independent experiments, mean ±SE. (**C**) MYC-CaP/CR and Pten-CaP/cE2 cells were treated with indicated concentrations of AUY922 for 48h. Adherent and non-adherent cells were collected and washed in 1x PBS. Cells were incubated with propidium iodide and cell death was assessed by flow cytometry. Each point represents mean ±SE of 3 independent experiments. * indicates p<0.05 compared to untreated control (0), by two-tailed t-test.

NVP-AUY922 (AUY922) is a resorcinlyic isoxazole amide (Fig. 1A) demonstrated to be a potent inhibitor of Hsp90 [16]. AUY922 is a non-geldanamycin analog, and does not exhibit the limitations of the geldanamycin analogs, therefor making this agent more promising for clinical testing. AUY922 mediates its effect by binding the ATPase domain of the Hsp90 N-terminus to inhibit the chaperone function of Hsp90. This action leads to the proteosomal degradation of several cancer relevant client proteins [16], including AR [17]. AUY922 has demonstrated anti-tumor activity in a variety of solid tumor pre-clinical mouse models, including PC3 prostate cancer cell and xenografts [16], [18]. Most recently, new generation Hsp90 inhibitors, including AUY922 have been reported to achieve biologic responses in prostate cancer cell lines and human prostate cancer tissue when compared to the geldanamycin analog 17-AAG [17]. Phase I and II studies in hematologic malignancies and solid tumors, including breast cancer and multiple myeloma, are ongoing with AUY922.

The objective of this study was to test the ability of AUY922 to antagonize the transcriptional and/or protein stability of the AR in castrate resistant MYC-CaP/CR and Pten-CaP/cE2 prostate cancer cell lines as well as a transplant mouse model recently developed in our laboratory [19], [20]. Further, we wanted to investigate the antitumor and therapeutic efficacy of AUY922 as a single agent and in combination with docetaxel chemotherapy.

## Materials and Methods

### Ethics Statement

The Institute Animal Care and Use Committee (IACUC) at Roswell Park Cancer Institute (RPCI) approved all mouse protocols used in this study. Our approval/protocol ID is 1137M.

### Cell culture and reagents

#### Isolation of a castrate resistant MYC-CaP cell line

The MYC-CaP/CR cell line was generated from our previously reported MYC-CaP castrate resistant transplant tumor model [19]. MYC-CaP/CR tumor pieces (∼4 mm^2^) were placed in a 6 well culture plate and removed after being cultured for 24 hours in supplemented DMEM high glucose media (10% FBS; 1% penicillin/streptomycin). Adherent cells were a mixed population of MYC-CaP/CR tumor cells and fibroblasts. These cells were cultured until approximately 80% confluent. Serial passaging of these heterogeneous cultures was performed, until a homogeneous monolayer of MYC-CaP/CR cells was present. MYC-CaP/CR cell lines were subsequently cultured in DMEM medium (Gibco) supplemented with 10% fetal bovine serum and 1% penicillin/streptomycin at 37°C, 5% CO_2_. Pten-CaP/cE2cE2 cells were a kind gift from Dr. Roy-Burman (University of California, Los Angeles) and cultured in DMEM medium (Gibco) supplemented with 10% fetal bovine serum and 1% penicillin/streptomycin at 37°C, 5% CO_2_. PC3 cells were obtained from the American Type Culture Collection (ATCC) and maintained in RPMI 1640 media (Gibco) supplemented with 10% fetal bovine serum and 1% penicillin/streptomycin at 37°C, 5% CO_2_.

For *in vitro* experiments, NVP-AUY922 (Novartis) was dissolved in dimethyl sulfoxide (DMSO) for the preparation of stock solutions (10 mM). The synthetic androgen, methyltrienolone (R1881; Sigma-Aldrich), was dissolved in ethanol for the preparation of stock solutions (10 mM). The proteasome inhibitor, MG132 (Sigma-Aldrich), was dissolved in DMSO for the preparation of stock solutions (10 mM). The translation inhibitor, cycloheximide (Sigma-Aldrich), was dissolved in ethanol for the preparation of stock solutions (5 mg/ml). Antibodies used for immunoblotting and/or immunohistochemistry (IHC) were anti-androgen receptor (Santa Cruz), GAPDH (Cell Signaling), c-MYC (Epitomics) and activated caspase 3 (Cell Signaling). For *in vivo* studies, docetaxel was obtained from the Roswell Park Cancer Institute pharmacy and diluted to 1 mg/ml in PBS before administration to animals. NVP-AUY922 was dissolved in 5% dextrose in distilled water (D5W) at a concentration of 4 mg/ml.

### Cell growth and cell death assays

MYC-CaP/CR or Pten-CaP/cE2 cells (4×10^4^/ml) were left to adhere overnight in 24 well plates (BD Biosciences) and incubated with indicated concentrations of NVP-AUY922 for 24 and 48 hours. Cell growth was measured by fixation and staining of adherent cells with 10% methanol in crystal violet for 30 minutes. Stained cells were made soluble in absolute methanol and absorbance was detected at an emission length of 570 nm. Viability (cell death) was measured by incubating adherent and non-adherent cells with 1 µg/ml propidium iodide (Sigma-Aldrich) uptake and quantitated with a FACS Caliber flow cytometer.

### Western blot

MYC-CaP/CR or Pten-CaP/cE2 cells were washed in PBS and lysed in RIPA buffer (Sigma-Aldrich) containing 1× protease and phosphatase inhibitors (Sigma-Aldrich). Equal amounts of protein were separated by electrophoresis using 4–15% SDS-PAGE gradient gels (Bio-Rad) and protein was transferred to nitrocellulose membranes (Biometra). Secondary HRP conjugated antibodies were from Dako. Detection was carried out using chemiluminescence reagents (PerkinElmer).

### Androgen receptor transcription activity

The androgen responsiveness status of MYC-CaP/CR cells was determined using a commercially available lentiviral-based luciferase reported kit (Cignal Lenti AR Reporter (Luc) kit; SABioscience) according to manufactures instructions.

### Quantitative real-time PCR

Total RNA was extracted by TRIzol (Invitrogen) according to manufactures instructions. One microgram of RNA was used to perform cDNA synthesis by iScript cDNA synthesis kit (Bio-Rad). One microliter of cDNA synthesis reaction was then subjected to PCR amplification by using iQ SYBR green kit (Bio-Rad). PCR signals were recorded and analyzed by Bio-Rad CFX Connect real-time PCR detection system. The sequences of primers are: FKBP5 forward, 5′ GCCGACTGTGTGTGTAATGC 3′, and reverse, 5′ CACAATACGCACTTGGGAGA 3′; GAPDH forward, 5′ GTCTTCACCACCATGGAGAAG 3′, and reverse, 5′CAAAGTTGTCATGGATGACCTTGG 3′. ΔΔCt values were calculated and used to determine fold changes of mRNA.

### Histology/Immunohistochemistry

Mice were sacrificed by CO_2_ asphyxiation at defined time points. Tumor tissue was fixed in 10% buffered formalin overnight followed by an additional 24 hours in 70% ethanol. For antigen retrieval, slides were boiled for 10 minutes in 10 mM sodium citrate pH 6 solution for all antibodies. ImmPRESS detection system (Vector Laboratories) was used for detection of all primary antibodies. Staining was visualized using 3,3′-Diaminobenzidine (DAB) (Sigma, Saint Louis, MO, FAST 3,3′-Diamino benzidine) and slides were counterstained with hematoxylin. For quantitation of IHC staining representative images (3–6) were obtained using a Zeiss light microscope (Zeiss). Positive nuclear staining for c-MYC and activated caspase 3 was quantified by Aperio ImageScope (v11.1.2.760).

### *In vivo* animal studies

The Institute Animal Care and Use Committee at Roswell Park Cancer Institute approved all mouse protocols used in this study. Mice were housed in an animal facility maintained on a 12-h light/dark cycle, at a constant temperature (22±2°C) and relative humidity (55±15%). Tap water and food were available *ad libitum*. FVB male mice (4–6 weeks old) were purchased from NCI Frederick (Maryland, USA). SCID mice were purchased from an in house colony maintained at Roswell Park Cancer Institute. All mice were surgical castrated up to 10 days before tumor inoculation. Surgical castration was performed by shaving the surgical site. An incision was made in the scrotum and in the tunica of the testicles with scissors. The testis, vas deferens and attached testicular fat pad were removed through the incision site. The incision site will be closed with surgical staples. The LuCaP23 androgen independent (AI) xenograft model [21] was a generous gift from Dr Robert Vessella (University of Washington, Seattle WA). This model was maintained by serial passaging of tumor tissue to pre-castrated SCID male mice.

#### Therapy studies

Pre-castrated male FVB male mice were inoculated with MYC-CaP/CR cells (1×10^6^) suspended in PBS by subcutaneous injection. Likewise, pre-castrated male SCID mice were inoculated with PC3 (1×10^6^) cells suspended in PBS by subcutaneous injection, or implanted with a LuCaP 23 AI tumor piece (4 mm^2^) subcutaneously. For docetaxel single treatment studies, tumor bearing mice received docetaxel therapy (10–50 mg/kg once weekly; i.p. injection). For combination studies, tumor bearing animals received vehicle (D5W), docetaxel (10 mg/kg, once weekly; i.p. injection), NVP-AUY922 (40 mg/kg, 5 days on 2 days off; i.p. injection) or combination. In all studies, mice were weighed weekly to monitor for toxicity. Tumor growth was assessed by serial caliper measurements twice weekly.

### Statistical Analysis

Data are displayed as the mean ± SE. Differences were determined using one or two-way ANOVA and two-tailed paired t tests where indicated, using GraphPad Prism software. P values less than 0.05 were assigned statistically significant.

## Results

### Response of castrate resistant prostate cancer cells to AUY922 treatment *in vitro*

MYC-CaP castrate resistant (MYC-CaP/CR) and Pten-CaP castrate resistant (Pten-CaP/cE2) cells were exposed for 48 hours to increasing concentrations of AUY922. As shown in Fig. 1B, low nanomolar concentrations of AUY922 were sufficient to inhibit the growth of MYC-CaP/CR and Pten-CaP/cE2 cells. Further, AUY922 induced a dose dependent increase in cell death of MYC-CaP/CR and Pten-CaP/cE2 cells at concentrations of 50–500 nM based on PI uptake analysis (p<0.05; Fig. 1C).

### AUY922 induces proteasome degradation of AR and inhibits AR transcriptional activity in MYC-CaP/CR and Pten-CaP/cE2Pten-CaP/cE2 cells

**Figure 2:**
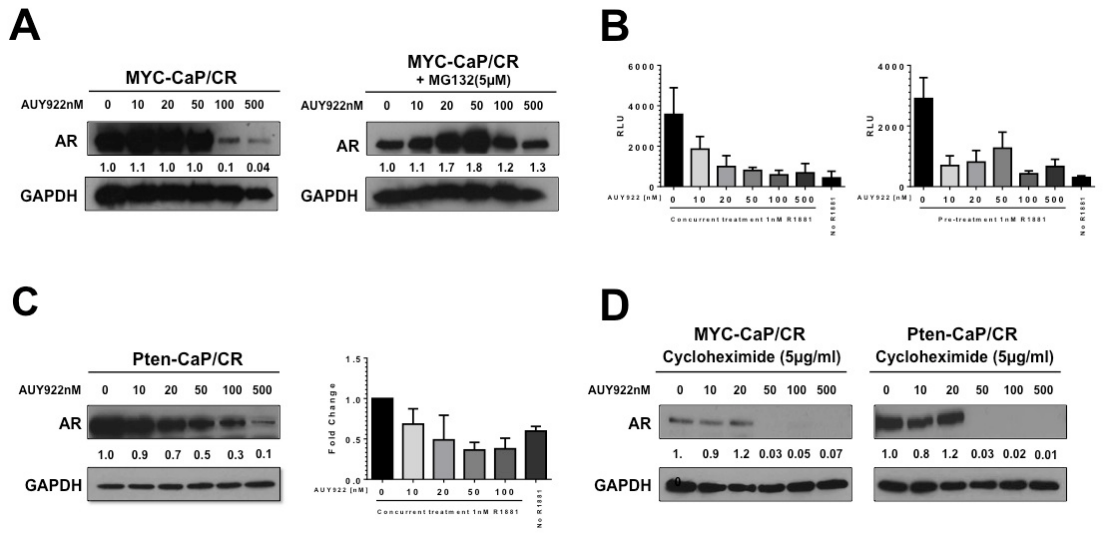
AUY922 effects on AR expression and activity in castrate resistant prostate cancer cells. (**A**) MYC-CaP castrate resistant cell were treated for 48 hr with increasing concentrations of AUY922 or increasing concentrations of AUY922 concurrently with the proteasome inhibitor MG132 [0.5 µM]. Expression of AR protein was assessed by western blot. (**B**) MYC-CaP/CR cell lines with stable transfection of an AR reporter plasmid (ARE-Luc) were incubated in androgen depleted cell culture conditions for 6 hours. Cells were treated concurrently with 1 nM R1881 and indicated concentrations of AUY922 overnight or pre-treated with 1 nM R1881 for 4 hr before adding the indicated concentrations of AUY992 Luminescence intensity was measured and quantitated. Columns represent 3 independent experiments; mean ±SE. (**C**) Pten-CaP/cE2 cells were treated with increasing concentrations of AUY922 for 48 hr. Expression of AR protein was assessed by western blot. Transcriptional activity of AR was performed by measuring FKBP5 mRNA expression level. Cells were incubated in androgen depleted cell culture conditions for 6 hours, and then treated concurrently with 1 nM R1881 and indicated concentrations of AUY922 overnight. Experiments represent 3 independent experiments, mean ±SE. (**D**) MYC-CaP/CR and Pten-CaP/cE2 cells were treated concurrently with 5 µg/ml cycloheximide and increasing concentrations of AUY922. Expression of AR protein was assessed by western blot. GAPDH served as protein loading control. The densitometry was performed by image j analysis. The numbers were shown below the bands relative to the control cells.

It is documented that Hsp90 inhibition often results in the proteasome degradation of its client proteins [16]. We therefor wanted to determine AR protein stability and/or transcriptional activity was attenuated by AUY922 in our castrate resistant models. MYC-CaP/CR and Pten-CaP/cE2 cells treated with increasing concentrations of AUY922 for 48 hours demonstrated a dose dependent loss of AR protein, most noticeable at concentrations of 100 nM and 500 nM (Fig. 2A and 2C). Concurrent treatment with AUY922 and the proteasome inhibitor, MG132 (0.5 µM), confirmed that AUY922 mediated loss of AR protein was by proteasome degradation (Fig. 2A). The effect of AUY922 on AR transcriptional activity was assessed by using MYC-CaP/CR cells with stable expression of a luciferase reporter driven by androgen response elements (ARE-Luc). MYC-CaP/CR/ARE-Luc cells were incubated with increasing concentrations of AUY922 and 1 nM R1881 concurrently overnight or pretreated with 1 nM R1881 for 4 hours followed by increasing concentrations of AUY922 overnight. Due to the constitutive expression of luciferase in Pten-CaP/cE2 cells, we assessed the mRNA expression level of AR downstream gene, FKBP5, to investigate the effect of AUY922 on AR transcriptional activity. Our results demonstrate that AUY922 inhibits AR transcriptional activity at low nanomolar concentrations in both castrate resistant cell lines (Fig. 2B and 2C). Further, these results also indicate that AR protein degradation is not required for AUY922 to antagonize AR transcription. In addition, we utilized cycloheximide to block new protein synthesis to further suggest that low nanomolar AUY922 is able to reduce AR transcriptional activity without affecting AR protein level (Fig. 2D).

### Co-treatment of AUY922 and docetaxel increases cell death of MYC-CaP/CR and Pten-CaP/cE2 cells

**Figure 3:**
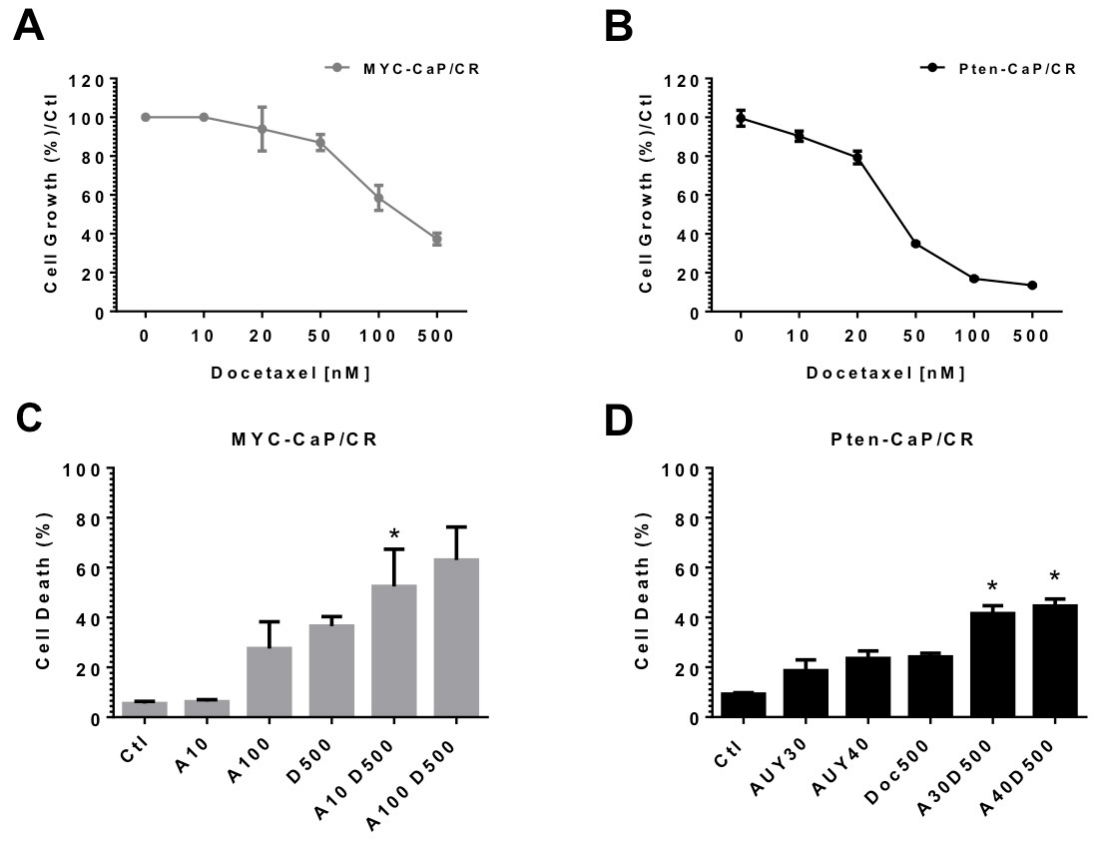
Response of castrate resistant prostate cancer cell lines to docetaxel treatment. Castrate resistant cell lines were treated with indicated concentrations of docetaxel and/or AUY922 for 48 h. MYC-CaP/CR (A) and Pten-CaP/cE2 (B) cell lines were treated with indicated concentrations of docetaxel for 48 h. Adherent cells were fixed with 70% EtOH. Cell growth was assessed by staining fixed cells with crystal violet and measuring absorbance at 570 nm. MYC-CaP/CR (C) and Pten-CaP/cE2 (D) cells were treated with indicated concentrations of AUY922 and/or docetaxel for 48 h. Cells were trypinized and washed in 1x PBS and incubated with propidium iodide (PI). Percentages of dead cells (PI positive cells) were determined by flow cytometry. Columns represent the mean ±SE, * indicates significantly greater in the combination compared to treatment with each drug alone (p = 0.03 as determined by one-way ANOVA).

We first investigated the sensitivity of MYC-CaP/CR and Pten-CaP/cE2 cells to the growth inhibition abilities of docetaxel. Drug treatment exhibited the ability to inhibit cell growth at low nanomolar concentrations in both cell lines (Fig. 3A–B).

We selected to treat MYC-CaP/CR cells for 48 hours with 10 nM AUY922 (a concentration where AR protein is not degraded but AR transcriptional activity is inhibited) and 100 nM AUY922 (a concentration where AR protein is degraded and AR transcriptional activity is inhibited) with 500 nM docetaxel. Fig. 3C shows that combination of 10 nM AUY922 with docetaxel significantly increases cell death compared to treatment with either drug alone. Combination of 100 nM AUY922 with docetaxel still increased cell killing compared to each single treatment though was not significant. Further, we treated Pten-CaP/cE2 cells with 30 and 40 nM AUY922 (concentrations of AUY922 that do not drastically effect AR protein expression) with 500 nM docetaxel, showing that these combination treatments of AUY922 and docetaxel are able to induce greater cell death compared to each single treatment (Fig. 3D). These data indicate that Hsp90 is potentially involved in the transcriptional activity of AR and that disrupting AR interaction with Hsp90 via its inhibition significantly antagonizes AR activity in MYC-CaP/CR and Pten-CaP/cE2 cells independent of AR protein expression, allowing for greater induction of cell death in combination with docetaxel.

### MYC-CaP/CR tumors are resistant to docetaxel therapy *in vivo*

**Figure 4:**
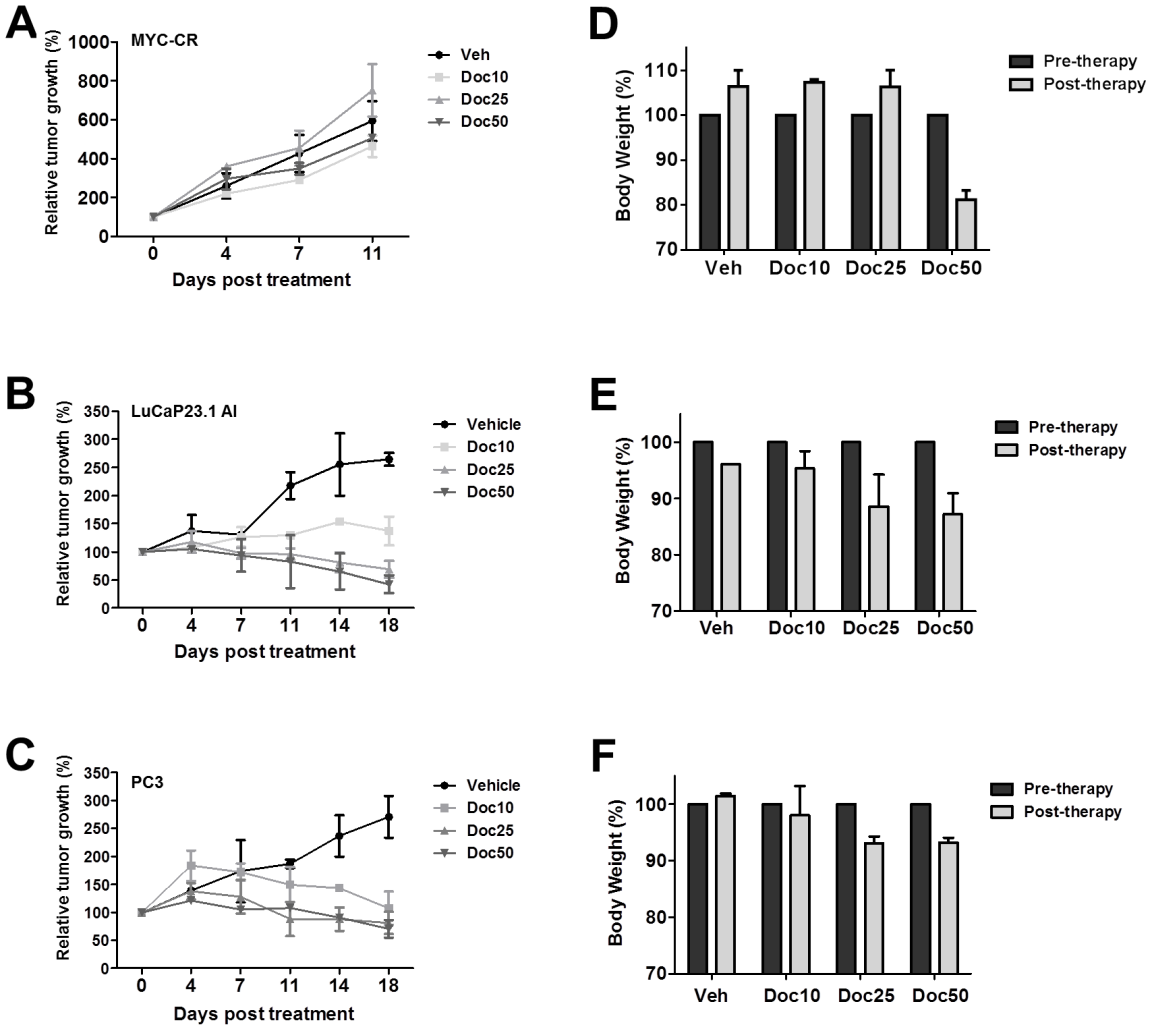
Docetaxel treatment of castrate resistant prostate cancer tumor models *in vivo*. (**A**) MYC-CaP/CR cells (5×10^6^), (**B**) LuCaP23.1 AI tumor chunk (∼5 mm^2^) and (**C**) PC3 cells (5×10^6^) were injected or placed subcutaneously into the flank of castrated male FVB (MYC-CaP/CR) or SCID (LuCaP23.1 AI and PC3) mice. Treatment was initiated when tumors measured approximately 50 mm^2^. Docetaxel was received from the pharmacy at RPCI as an aqueous solution (20 mg/ml) and diluted to 1 mg/ml in 1× PBS daily. Mice were treated with docetaxel doses of 10, 25 and 50 mg/kg weekly by intra-peritoneal (i.p.) injection for duration of 2 cycles (MYC-CaP/CR) or 3 cycles (LuCaP23.1 AI and PC3). Tumor growth was monitored by serial caliper measurements bi-weekly. Tumor size was calculated by L × W. All treatment groups consisted of 3–4 mice. Each treatment group was normalized to the pretreatment measurements and converted to percent tumor growth. Each point represents mean tumor size ±SE. (D–F) All mice were weighed 2 times/week to monitor docetaxel toxicity. Toxicity was considered to be occurring when mouse body weight was reduced ≥20%. Each column is represented by 3–4 individual mice/treatment and represented as percent bodyweight change compared to pre-treatment measurements, mean ±SE.

To assess the ability of docetaxel anti-tumor activity *in vivo* towards MYC-CaP/CR tumors, castrated FVB mice bearing MYC-CaP/CR tumors were treated with varying doses of docetaxel. As controls, we also treated castrated SCID mice bearing human xenograft tumors, LuCaP23.1 AI and PC3, which are known to respond to docetaxel therapy *in vivo*. Fig. 4B and 4C demonstrate that LuCaP23.1 AI and PC3 tumors were sensitive to docetaxel treatment. Further, each dose did not generate significant toxicity during treatment (Fig. 4E and 4F). In contrast, MYC-CaP/CR tumors were resistant to all doses of docetaxel treatment (Fig. 4A), and toxicity was indicated in mice treated with 50 mg/kg docetaxel (Fig. 4D).

### AUY922 decreases AR expression in MYC-CaP/CR tumors and combines with docetaxel to increase therapeutic efficacy

**Figure 5:**
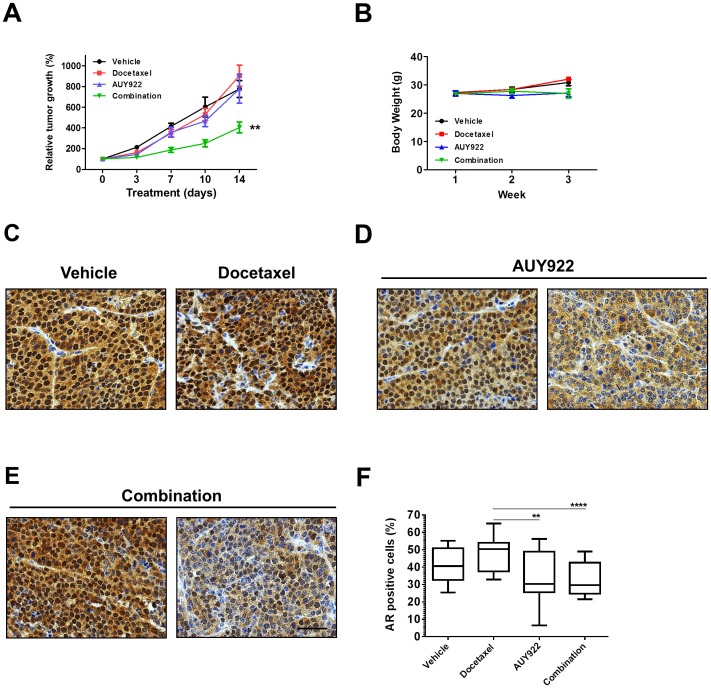
Combination of AUY922 and docetaxel result in increased anti-tumor activity and loss of AR expression in* vivo*. (**A**) MYC-CaP/CR cells (5×10^6^) were injected subcutaneously into the flank of castrated male FVB mice. Treatment was initiated when tumors measured approximately 50 mm^2^ (L × W). Mice were treated with vehicle (D5W, i.p., *n = 6*), docetaxel (10 mg/kg, weekly, i.p., *n = 7*), AUY922 (40 mg/kg, 5d on 2d off, i.p., *n = 5*) or combination (*n = 6*) for 2 weeks. Tumor size was monitored by serial caliper measurements biweekly. Tumor size was calculated by L × W. Each treatment group was normalized to the pretreatment measurements and converted to percent tumor growth. Each point represents mean tumor size ±SE. Two-way ANOVA: ** p = 0.009 docetaxel versus combination, p = 0.002 AUY922 versus combination. (**B**) Weekly bodyweight measurements indicate that therapy was not toxic. Each point represents mean ±SE. (**C–E**) Tumor tissue was collected at the conclusion of therapy, fixed in 10% normal buffered formalin and embedded in paraffin. Four micron (4 µM) sections of tumor tissue were assessed by immunohistochemistry for androgen receptor expression. Magnification (x40), scale bar = 500 µM. Positive nuclear staining for AR was quantified using aperio imagescope analysis of 6 random fields at x40 magnification. Scale bar = 500 µM. Unpaired two-tailed t-test with Welch’s correction: **** p<0.0001 combination versus docetaxel; ** p = 0.0016 docetaxel versus AUY922.

The anti-tumor activity of AUY922 and docetaxel *in vivo* was investigated by treating castrated FVB mice with MYC-CaP/CR tumors. Tumor bearing mice were treated with vehicle (D5W; 5d on 2d off), AUY922 (40 mg/kg i.p.: 5d on 2d off), docetaxel (10 mg/kg i.p.: once weekly) or combination for 2 cycles (14 days). No significant toxicity was observed in all therapy groups as shown by body weight measurements (Fig. 5B). As seen in Fig. 5A, compared to vehicle treatment, a two week treatment with AUY922 or docetaxel alone did not significantly reduce tumor growth. Notably, combination of AUY922 with docetaxel therapy significantly abrogated the growth of MYC-CaP/CR tumors compared to each single treatment (AUY922, p = 0.002; docetaxel, p = 0.009). Further, histochemical analysis of treated MYC-CaP/CR tumor tissue for AR expression revealed that both vehicle and docetaxel treated tumors displayed strong AR nuclear staining (Fig. 5C). Interestingly, AUY922 as a single treatment and in combination with docetaxel showed that Hsp90 inhibition had a heterogeneous effect overall on tumor AR expression. Fig. 5D and 5E show representative immunostaining of MYC-CaP/CR tumor sections, which demonstrate sections of tumor thatremained positive for AR nuclear expression, while adjacent sections of tumors exhibited loss of nuclear AR expression.

### AUY922 decreases AR transcriptional activity in MYC-CaP/CR tumors and combines with docetaxel to increase tumor cell death

**Figure 6:**
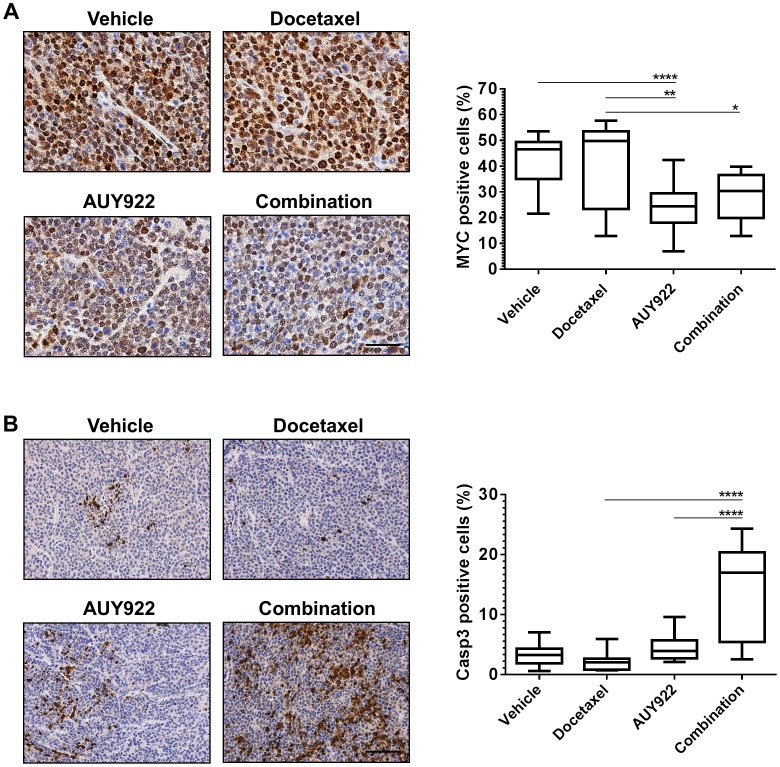
AUY922 and docetaxel combination therapy significantly attenuate AR transcriptional activity and increase tumor cell death *in vivo*. (**A**) Representative c-MYC immunostaining. Positive nuclear staining for c-MYC was quantified using aperio imagescope analysis of 6 random fields at x40 magnification. Scale bar = 500 µM. Two-tailed t-test: **** p<0.0001 AUY922 versus vehicle; ** p = 0.006 AUY922 versus docetaxel; * p = 0.03 combination versus docetaxel. (**B**) Representative cleaved caspase-3 immunostaining. Positive nuclear staining for cleaved caspase-3 was quantified using aperio imagescope analysis of 6 random fields at x20 magnification. Scale bar = 500 µM. Two-tailed t-test: **** p<0.0001 combination versus single treatments.

To assess the activity of AUY922 on the attenuation of transcriptional activity *in vivo*, we used immunostaining to determine the expression of the c-MYC transgene, which in this tumor model is driven by AR transcription via a probasin promoter [22], [23]. Docetaxel treatment did not induce a loss of c-MYC expression (42.3%) compared to vehicle treated tumors (41.2%; Fig. 6A), which was consistent with AR expression in tumor tissues. In contrast, tumors treated with AUY922, displayed significant reduction of c-MYC expression compared to vehicle treatment (42.3% verse 24.6%; p<0.0001) and docetaxel treatment (41.2% verse 24.6%; p = 0.006). Further, combination treatment exhibited similar loss of c-MYC expression compared with AUY922 single treatment, and was significant when compared to docetaxel single treatment (28.7% verse 41.2%; p = 0.03; Fig. 6A).

Tissues were next assessed for induction of cell death by immunstaining for cleaved caspase-3. A low level of cell death was observed in vehicle treated tissue (3.3%) with no significant increase in cell death following docetaxel (2.1%) or AUY922 (4.4%) single treatments. However, combination treatment significantly increased the number of cleaved caspase-3 positive tumor cells compared to docetaxel (14.5% verse 2.1%; p<0.0001) and AUY922 (14.5% verse 4.4%; p<0.0001) single treatments (Fig. 6B).

## Discussion

Docetaxel chemotherapy still remains a primary standard for care for patients with castrate resistant prostate cancer. Unfortunately, disease progresses despite docetaxel treatment or does not respond at all. Therefore, the identification of new drugs, which can enhance or sensitize patients to docetaxel chemotherapy, is greatly needed. Mechanisms underlying resistance to docetaxel have been demonstrated to be multifactorial in cell lines and clinical samples of different tumor types [24]. Identified mechanisms include decreased cellular accumulation due to efflux proteins, including P-glycoprotein (P-gp)/MDR1 and MDR2 [25] and mutations that inhibit docetaxel from directly binding β-tubulin [26]. In this study, we demonstrate that the second generation Hsp90 inhibitor AUY922 induces potent inhibition of AR transcription associated with antitumor activity, and increases cell death in combination with docetaxel in castrate resistant prostate cancer cell lines. Further, we show that AUY922/docetaxel combination therapy results in significant antitumor activity. This increase of therapeutic efficacy *in vivo* by AUY922/docetaxel combination was associated with attenuated AR expression and transcription and a significant increase of tumor cell death.

It has been previously reported that AUY922 can induce the loss of cell growth and increase cell death of prostate cancer cell lines [17]. Our data are consistent with this observation, as AUY922 treatment of the MYC-CaP/CR and Pten-CaP/cE2 cell lines resulted in a loss of cell growth an increase in cell death in a dose dependent manner. Further, AR is also a well-known client protein of Hsp90, and recently two investigations by Gandhi *et al* [10] and Centenera *et al* [17] demonstrated that Hsp90 inhibition by AUY922 resulted in loss of AR protein expression in the murine MYC-CaP/AS and human LnCaP prostate cell lines respectively. Our studies also demonstrated that AUY922 depleted AR protein expression in a dose dependent manner and was rescued by proteasome inhibition. We further observed that AUY922 treatment of MYC-CaP/CR and Pten-CaP/cE2 cells *in vitro* resulted in reduction in AR transcriptional activity. Interestingly, this mediated loss of AR transcriptional activity was independent of AR protein degradation. Previous data has alluded to the importance of Hsp90 in AR transcriptional activity *in vitro* [27] and Hsp90 regulation of AR transactivation in progression to castrate resistant prostate cancer following castration *in vivo* [28]. Our present study further supports the importance of Hsp90 interaction with AR transcriptional function in castrate resistant prostate cancer.

Although the Hsp90 inhibitor 17-AAG ultimately failed in initial phase I clinical trials in patients with advanced solid tumors [12], [14], [29], one patient with prostate cancer was reported to have achieved disease stabilization with a 25% reduction in PSA levels [30]. This result lead to a phase II trial of 17-AAG treatment in patients with advanced metastatic castrate resistant prostate cancer, though unfortunately no clinical responses were observed [31] leading to decreased enthusiasm in further clinical development of Hsp90 inhibitors for prostate cancer treatment. Despite these results, 17-AAG has been recently tested in the clinic with combination therapeutic strategies. In a Phase I study of 17-AAG in combination with docetaxel, 25% of patients with prostate cancer obtained a decline in PSA levels by ≥20% [32]. In our studies, we tested our MYC-CaP/CR and Pten-CaP/cE2 cell lines to sensitivity of docetaxel *in vitro*, and found that cells were sensitive to growth inhibition. Additionally, combination of AUY922 with docetaxel displayed greater induction of cell death. Further, our observed increases in cell death following combination treatment were associated with loss of AR transcription, but not AR protein degradation. This exciting result demonstrates that AUY922 inhibition of AR does not require protein degradation to increase sensitivity to docetaxel treatment.

Our *in vivo* studies initially revealed that MYC-CaP/CR tumors are resistant to docetaxel chemotherapy, when compared to docetaxel sensitive PC3 [33] and LuCaP23.1 AI [34] prostate tumor models. However, combination treatment of MYC-CaP/CR tumors with AUY922 and docetaxel significantly reduced tumor growth compared to each single treatment arm. Further, *in vivo* assessment of AR expression in MYC-CaP/CR tumor tissue revealed heterogeneous staining for AR within tumors treated with AUY922 as monotherapy or in combination. Sections of tumor tissue displayed loss of nuclear AR expression, whereas other sections remained with nuclear AR expression. Assessment of AR transcriptional activity in response to AUY922 treatment was determined by c-MYC immunostaining, as c-MYC within the MYC-CaP model is an AR dependent transgene [22], [23]. Excitingly, in line with our *in vitro* results, overall AUY922 inhibition did not require a loss of AR protein expression to reduce overall AR transcriptional activity. This loss of AR transactivation was associated with significant increase in tumor cell death in combination treatment, indicating AR inhibition alone with AUY922 was not enough of a catalyst to induce tumor cell death. This result is in contrast with Centenera *et al*, as their report demonstrated significant increase in tumor cell death by AUY922 single treatment [17]. The difference in these results may be explained by the different prostate cancer experimental models and the fact that Centenera *et al* treated androgen naïve post-prostatectomy tissue *ex vivo* allowing for greater exposure to AUY922.

To date, combination treatments with targeted therapies to increase the efficacy of docetaxel in the first-line setting have been unsuccessful. For example, clinical trials with docetaxel in combination with vascular endothelial growth factor or Src inhibitors have failed to demonstrate increased overall survival compared to docetaxel alone [35]. Our preclinical study provides a rationale for combining docetaxel with an Hsp90 inhibitor to increase sensitivity to this chemotherapy drug. The mechanism of action and toxicity profile of the second generation Hsp90 inhibitors make this combination a rational hypothesis to be tested clinically. The inhibitory effect on AR transcriptional activity by Hsp90 inhibition also provides an intriguing strategy for maintenance therapy for continuous inhibition of AR signaling and potential combinations with other AR targeted therapies.

In conclusion, targeting AR signaling in the pre and post-docetaxel clinical setting has proven to provide benefit to patients; however targeted therapies with alternative mechanism of action to overcome or delay drug resistance are still needed. In this study, we show that the use of an Hsp90 inhibitor achieves inhibition of AR expression and transcriptional activity, which allows for sensitization to docetaxel chemotherapy and greater anti-tumor activity through the induction of tumor cell death. Due to the greater potential of targeting oncogenic signaling pathways by Hsp90 inhibition and the availability of new and more effective agents targeting this chaperone protein, further clinical investigation of Hsp90 inhibitors in rational combination strategies with already FDA approved therapies for castrate resistant prostate cancer is warranted.
